# CD1a expression by Barrett's metaplasia of gastric type may help to predict its evolution towards cancer

**DOI:** 10.1038/sj.bjc.6602415

**Published:** 2005-03-08

**Authors:** F Cappello, F Rappa, R Anzalone, G La Rocca, G Zummo

**Affiliations:** 1 Human Anatomy Section, Department of Experimental Medicine, University of Palermo, Via alla Falconara 120, 90136 Palermo, Italy

**Keywords:** Barrett's metaplasia, CD1a, carcinogenesis

## Abstract

As emerging in the recent literature, CD1a has been regarded as a molecule whose expression may reflect tumour evolution. The aim of the present work was to investigate the expression of CD1a in a series of Barrett's metaplasia (BM), gastric type (GTBM), with and without follow-up, in order to analyse whether its expression may help to diagnose this disease and to address the outcome. Indeed, GTBM may be confused sometimes with islets of ectopic gastric mucosa and its evolution towards dysplasia (Dy) or carcinoma (Ca) could not be foreseen. We showed a significant higher expression of CD1a in GTBM than in both Dy and Ca; nevertheless, the number of positive GTBM was significantly lower in the group of cases that at follow-up underwent Dy or Ca. Our data address that CD1a may be a novel biomarker for BM and that its expression may help to predict the prognosis of this pathology.

CD1a is a surface glycoprotein of 43–49 kDa that has been shown to be expressed by dendritic cells (DCs), cortical thymocytes and Langerhans cells of the skin (Dezutter-Dambuyant *et al*, 1990; Krenacs *et al*, 1993; Gregory *et al*, 2000). Moreover, the research of CD1a is commonly used to differentiate various cutaneous T-cell lymphomas from B-cell lymphomasand pseudolymphomas (Arai *et al*, 1999; Schmuth *et al*, 2001).

The antitumoral role of CD1a was recently proposed (Coventry and Morton, 2003a; Coventry and Heinzel, 2004; La Rocca *et al*, 2004). We also recently described that CD1a could be expressed in metaplastic epithelium of Barrett's oesophagus, both gastric and intestinal types, while normal gastric and colonic mucosa were negative to this marker (Cappello *et al*, 2003). In particular, we postulated that this marker may be useful in diagnosing Barrett's metaplasia (BM) and we hypothesised that its expression could predict a favourable outcome of this disease (Cappello, 2004).

Indeed, BM may evolve towards dysplasia (Dy) or carcinoma (Ca) (Cameron and Carpenter, 1997; Malhi-Chowla *et al*, 2000; Moreto, 2003). Nevertheless, to date, we do not have any marker that could help to predict BM evolution.

The aim of the present work was to detect CD1a expression in a large series of BM, Dy and Ca at the time of diagnosis and at follow-up. We would verify the diagnostic role of CD1a for BM and confirm our hypothesis concerning its prognostic role.

## MATERIALS AND METHODS

### Sample collection

We selected retrospectively from our files, 222 cases as follows: 166 specimens were BM of gastric type (GTBM) that underwent follow up; moreover, we selected 37 specimens of Dy and 19 of Ca that did not undergo follow-up. Finally, as control group, we selected 10 specimens from normal gastric mucosa. Sample collection was performed according to ethical standards. In addition, we collected, from the 166 cases of GTBM, the specimens that underwent follow-up, commonly between 12 and 36 months from first diagnosis; in 134 cases, the diagnosis of BM was confirmed (FU-GTBM), while in 23 cases, GTBM evolved towards dysplasia (FU-Dy) and in nine cases towards carcinoma (FU-Ca).

### Immunohistochemistry

We subjected all specimens of both groups to immunohistochemistry for CD1a, using a monoclonal antibody (DAKO, clone O10, 1 : 50) revealed by an avidin–biotin system (DAKO – LSAB2). In detail, 5 *μ*m sections were subjected to immunohistochemistry in triplicate, in order to minimize false-positivity errors. Nonimmune sera were substituted for negative controls and appropriate positive controls were run concurrently. 3-3′-Diaminobenzidine (DAB chromogen solution, DAKO, cat. no. K3467) was used as develop chromogen. A nuclear counterstaining with haematoxylin (DAKO, cat. no. S2020) was finally performed. The immunostainings were valued from two independent observers (FC and FR).

### Statistical analysis

For all groups of patients, data regarding positivity to CD1a expression were plotted using MS Excel software. Statistical analysis was performed using the Mann–Whitney *U*-test in order to verify the presence of a significant difference between CD1a-positive and -negative cases of GTBM that had undergone follow-up. In all cases, data were considered significant for values of *P*<0.05.

## RESULTS

### Immunohistochemistry

Thin sections of oesophageal specimens from all subjects were subjected to immunohistochemical analyses in order to evaluate CD1a expression in both metaplastic and dysplastic/cancerous tissues.

**Figure 1:**
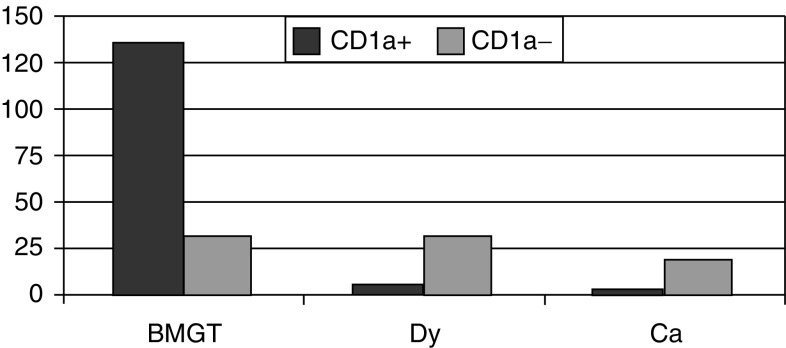
Distribution of specimens with respect to CD1a positivity at the time of diagnosis.

As summarised in Figure 1, the frequency of expression of CD1a was a distinguishing feature between metaplastic and dysplastic/carcinomatous lesions. In particular, the group of GTBM presented a higher number of CD1a-positive cases (166 out of 222, 81%), while this marker was found rather infrequently in both Dy (37 out of 222, 13.5%) and Ca (19 out of 222, 5.3%) groups.

**Figure 2:**
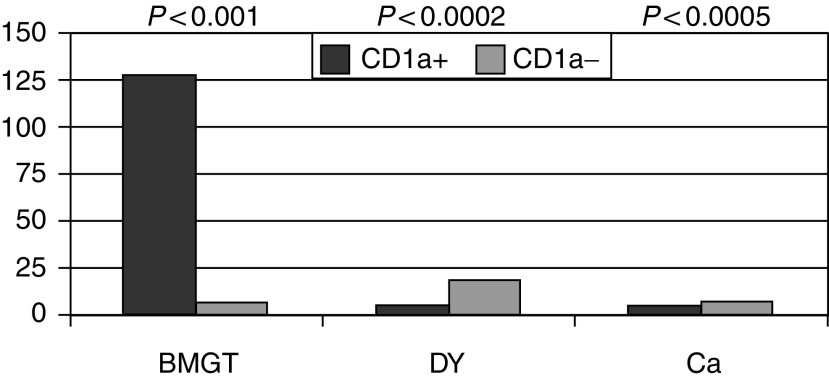
Diagrams of follow-up analysis for the group of patients (*n*=166) diagnosed for BM (BMGT). As shown, 80.7% of lesions (*n*=134) were confirmed as BMGT, 13.9% (*n*=23) developed Dy, while 5.4% (*n*=9) developed Ca. Lesions of the group originally classified as CD1a− were significantly more prone to evolve to Dy (*P*<0.0002) or Ca (*P*<0.0005) with respect to the CD1a+ group.

The aim of the present study was also to evaluate the usefulness of CD1a expression as a marker of evolution of the metaplastic lesions, as metaplasia has been regarded as a predisposing condition for the development of oesophageal Ca (Malhi-Chowla *et al*, 2000; Moreto, 2003). To this end, as shown in Figure 2, most of FU-Dy and FU-Ca cases evolved from the group of subjects that at the time of the first diagnosis featured the absence of CD1a protein. Statistical analysis showed a significant difference between FU-GTBM (*P*<0.001), FU-Dy (*P*<0.0002) and FU-Ca (*P*<0.0005) comparing CD1a+ and CD1a− groups.

**Figure 3:**
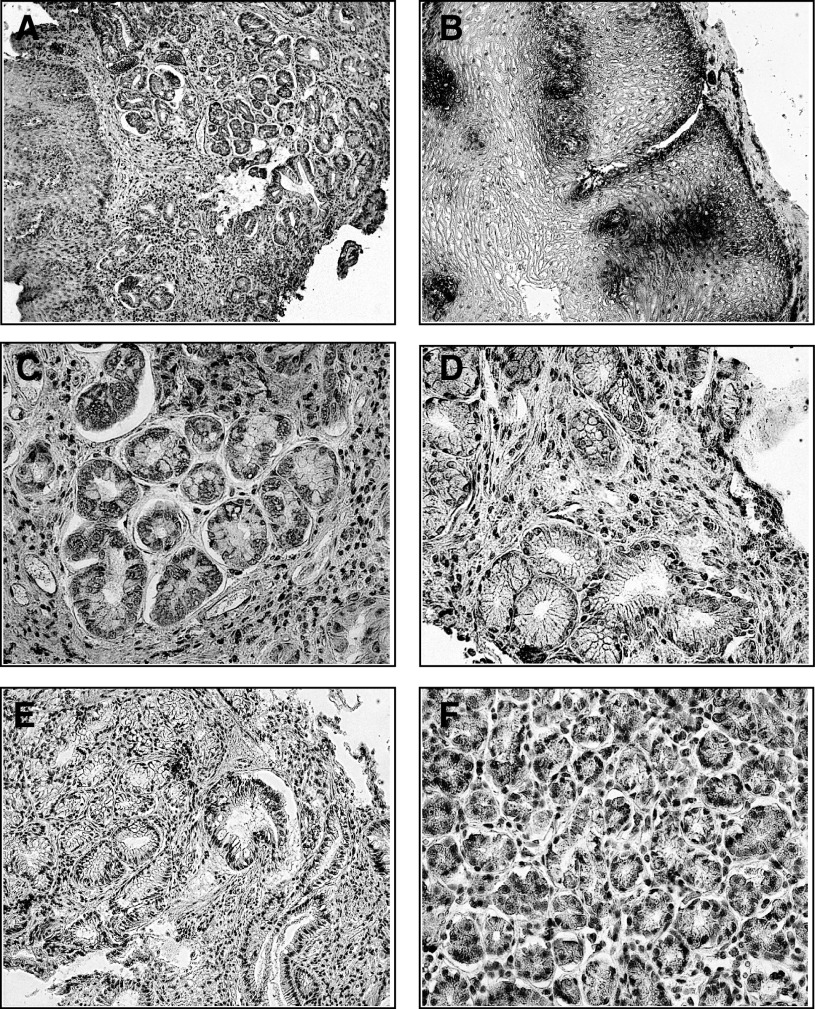
Panel of immunohistochemistry microphotographs showing a biopsy for BM (**A**) with positive CD1a dendritic elements between epithelial cells (**B**), as well as positive epithelial cells at the level of metaplastic glands (**C**). By contrast, CD1a was absent in dysplastic (**D**) and neoplastic (**E**) glands as well as in normal gastric mucosa (**F**).

Finally, Figure 3 shows immunohistochemical results. Positivity for CD1a in BM specimens was present not only at the level of intraepithelial DCs (Figure 3A and B), but above all in metaplastic epithelium (Figure 3A and C), while only few scattered stromal elements were found between metaplastic glands (Figure 3C). Moreover, normal gastric mucosa (Figure 3F) was always negative for CD1a, as well as most of the cases of Dy (Figure 3D) and Ca (Figure 3E).

## DISCUSSION

CD1a is usually expressed by immature DCs (Arrighi *et al*, 2003). Its expression by APC was recently supposed to be correlated to a better prognosis in breast cancers (Coventry and Morton, 2003b; Poindexter *et al*, 2004; Thomachot *et al*, 2004). In particular, its overexpression by axillary lymph nodes could prevent lymph node metastases (Poindexter *et al*, 2004); nevertheless, the authors of this work found a higher number of mature CD83-positive DCs, but not of immature CD1a-positive DCs, in tumour-free sentinel lymph nodes than those containing tumours. In addition, its expression by epithelial cells of *in situ* ductal Ca of the breast was also suggested (Coventry and Heinzel, 2004). Indeed, the density of CD1a^+^ DCs within human tumours has been already associated with longer survival.

Barrett's oesophagous is an acquired metaplastic condition that results by a phenotypic switch of undifferentiated epithelial elements present in oesophageal mucosa (Fahmy and King, 1993; Chandrasoma *et al*, 2001). Barrett's metaplasia of gastric type is characterised by the presence of columnar cells resembling the gastric foveolar ones (Zwas *et al*, 1986). Although the presence of a severe inflammation was recently associated to a higher possibility to progress to cancer (Fitzgerald *et al*, 2002), the lamina propria surrounding metaplastic epithelium shows commonly a mild inflammatory infiltrate (Lee, 1999). Indeed, until now, the Dy arising on BM is the only well-established preneoplastic lesion of the oesophageal adenocarcinoma (Menke-Pluymers *et al*, 1993; Clark *et al*, 1996).

We already showed, in a smaller series, that CD1a may be expressed by epithelial cells of BM, both gastric and intestinal types (Cappello *et al*, 2003). In particular, we postulated a diagnostic role of this marker and supposed that it may also be useful to predict prognosis. Moreover, we already hypothesised a proimmunitary role of CD1a expression by epithelial cells (La Rocca *et al*, 2004), in accordance to Coventry and Heinzel (2004).

In the present work, we demonstrated that CD1a may have a diagnostic role for BM. In particular, the expression of CD1a by metaplastic epithelial cells might help to distinguish GTBM from the presence of ectopic gastric epithelium in the oesophageal mucosa, since gastric epithelium resulted negative. Moreover, our data strongly suggest a prognostic role of the expression of CD1a on GTBM; indeed, the number of FU-Dy and FU-Ca cases was significantly higher in the CD1a− GTBM group than in the CD1a+ GTBM group.

In our opinion, the present results may confirm that increased CD1a expression not only in DCs but above all in epithelial cells may prevent tumour progression, for instance regarding the evolution of GTBM towards Ca. The expression of CD1a on metaplastic epithelial cells might be induced by local humoral factors, like inflammatory cytokines, as well as other unknown stimulatory molecules.

In conclusion, our hypotheses concerning the diagnostic and prognostic role of CD1a for BM may be confirmed by the present work. Moreover, we suppose that CD1a expression may have an antitumoral role in BM mucosa, and also, as postulated, in neoplastic diseases arising at other anatomic sites (Coventry and Morton, 2003a; Coventry and Heinzel, 2004; Poindexter *et al*, 2004). As a future objective, epithelial CD1a+ cells interaction with DCs or T cells remains to be further investigated. At this stage, the possible explanation for the role of CD1a ectopic expression by epithelial elements remains unclear.

Regarding the intercellular signalling strategies, we suppose that DCs induce tumour cells apoptosis following the production of certain extracellular cytokines, as already postulated (Joo *et al*, 2002). It may be assumed that this is only one of the processes that take place following DCs activation. Considering the importance of intercellular signalling, mediated by both soluble factors and extracellular matrix-bound molecules, a future goal could be to determine the cytokine expression pattern of Barrett's metaplastic cells, and by what means it could modulate antitumour immune response. Indeed, although BM commonly shows a mild inflammatory infiltrate, we will consider now with great interest the investigation of the cellular features and molecular pattern of these immunitary cells.
